# FastDup: a scalable duplicate marking tool using speculation-and-test mechanism

**DOI:** 10.1093/bioinformatics/btaf633

**Published:** 2025-12-01

**Authors:** Zhonghai Zhang, Yewen Li, Ke Meng, Chunming Zhang, Guangming Tan

**Affiliations:** Institute of Computing Technology, Haidian District, Beijing 100190, China; School of Computer Science, University of Chinese Academy of Sciences, Haidian District, Beijing 100049, China; The Hong Kong University of Science and Technology, Sai Kung District, New Territories, Hong Kong 999077, China; Institute of Computing Technology, Haidian District, Beijing 100190, China; Institute of Computing Technology, Haidian District, Beijing 100190, China; Institute of Computing Technology, Haidian District, Beijing 100190, China; School of Computer Science, University of Chinese Academy of Sciences, Haidian District, Beijing 100049, China

## Abstract

**Summary:**

Duplicate marking is a critical preprocessing step in gene sequence analysis to flag redundant reads arising from polymerase chain reaction amplification and sequencing artifacts. Although Picard MarkDuplicates is widely recognized as the gold-standard tool, its single-threaded implementation and reliance on global sorting result in significant computational and resource overhead, limiting its efficiency on large-scale datasets. Here, we introduce FastDup: a high-performance, scalable solution that follows the speculation-and-test mechanism. FastDup achieves up to 20× throughput speedup with 32 threads and guarantees 100% identical output compared to Picard MarkDuplicates.

**Availability and implementation:**

FastDup is a C++ program available from Zenodo https://zenodo.org/records/15727829, Bioconda https://anaconda.org/bioconda/fastdup and GitHub https://github.com/zzhofict/FastDup.git under the MIT license.

## 1 Introduction

Duplicate reads are a pervasive issue in next-generation sequencing (NGS) data and arise primarily due to two sources: polymerase chain reaction (PCR) amplification during library preparation and sequencing artifacts such as optical duplicates. These duplicate reads can skew downstream analyses by inflating read counts, distorting allele frequency estimates, and potentially leading to false positive variant calls. Consequently, identifying the duplicate reads is a critical preprocessing step in gene sequence analysis.

Several tools have been developed to address this problem. Among these, Picard MarkDuplicates (GitHub Repository: https://github.com/broadinstitute/picard). Broad Institute (2025) has long been recognized as the gold standard due to its robust duplicate identification algorithm. However, its reliance on position-sorted data and single-threaded implementation limits its performance, especially for large-scale datasets. GATK MarkDuplicatesSpark (Van der Auwera and O’Connor 2020) and Sambamba (Tarasov *et al.* 2015) use the same duplicate identification algorithm as Picard MarkDuplicates but achieve better performance by leveraging parallel processing. However, they still depend on the global sorting results of genomic coordinates of all reads, which requires either a significant memory footprint or writing intermediate results to disk, thereby constraining their overall efficiency.

Streaming-based approaches, such as Samblaster (Faust and Hall 2014) and streammd (Leonard 2023), have been proposed to accelerate duplicate marking by eliminating the need for position-sorted reads and enabling direct piping after the mapping process. Samblaster uses a hash-based structure, while streammd utilizes a Bloom filter to identify duplicates in real time. However, both methods simplify duplicate marking by retaining only the first read and treating all subsequent reads with the same genomic coordinate as duplicates, a strategy that can compromise accuracy in scenarios where more nuanced differentiation is required.

To overcome these limitations, we present FastDup, a high-performance duplicate marking tool that combines the robustness of Picard’s duplicate identification algorithm with innovative performance optimizations. FastDup uses a novel speculation-and-test mechanism, wherein potential duplicates are speculated in parallel and subsequently verified in a streamlined testing phase. This approach is integrated into a fully parallelized pipeline that avoids global sorting while maintaining identical accuracy compared to Picard MarkDuplicates (Broad Institute 2025). Experiment results demonstrate that FastDup achieves over a 20× speedup compared to traditional methods, offering a scalable and efficient solution for high-throughput sequencing workflows.

## 2 Description

FastDup is implemented in C++ and leverages the htslib (Bonfield *et al.* 2021) for high-performance parsing of SAM/BAM files. To maximize I/O efficiency, it uses an asynchronous I/O pipeline that decouples file decompression/compression from computational tasks, enabling simultaneous multi-threaded read and write operations through lock-free queues. Similar to Picard MarkDuplicates (Broad Institute 2025), FastDup adopts a two-pass duplicate marking strategy to accurately identify all duplicates while retaining the highest-scoring read pairs based on the same scoring system.

**Figure 1.**
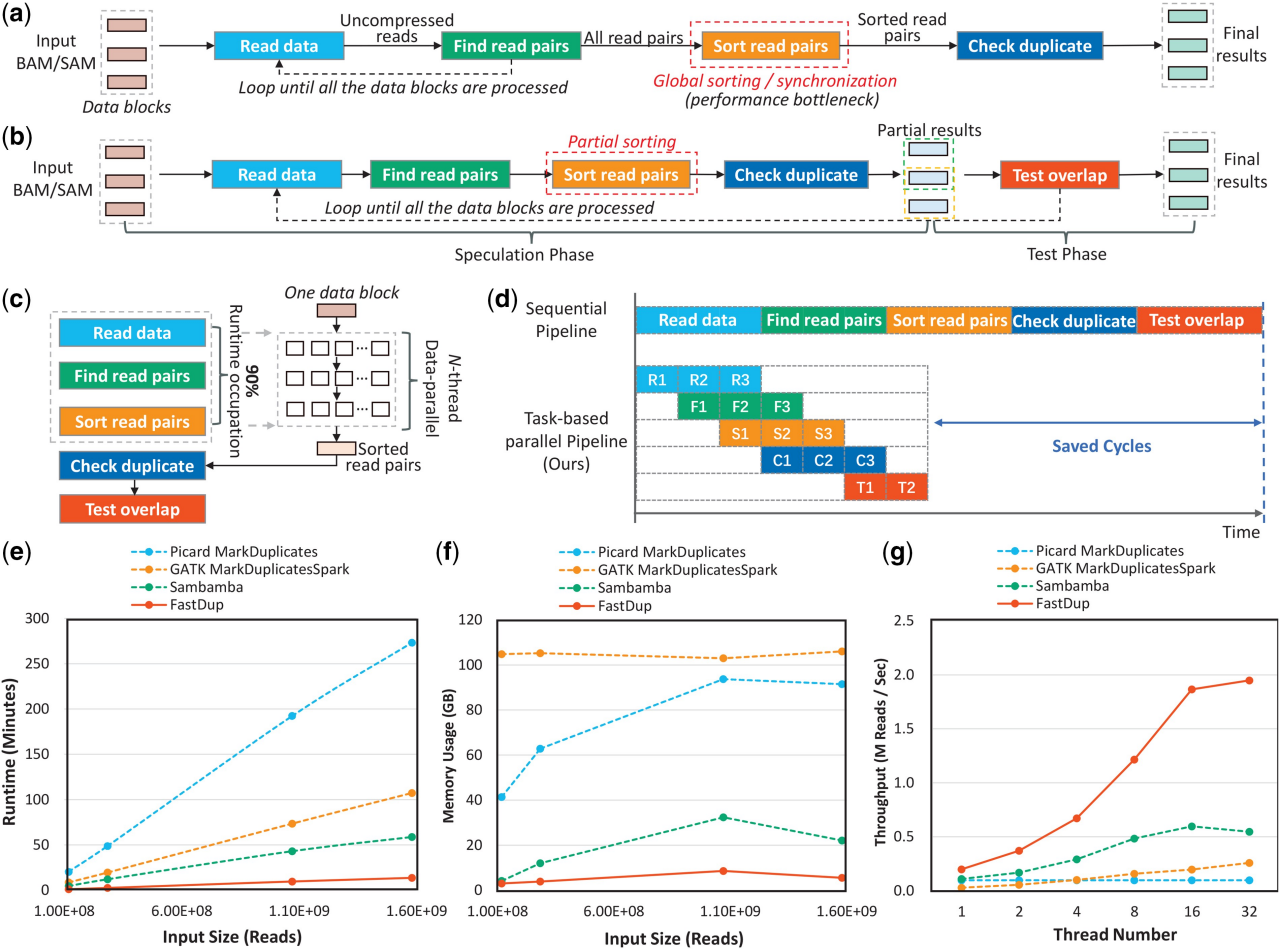
(a) The workflow of marking duplicate in Picard. (b) The workflow of speculation-and-test strategy in FastDup. (c) FastDup accelerates the workflow using data parallelism. (d) The overall workflow is further optimized by using a task-parallelism strategy. (e) Picard MarkDuplicates, GATK MarkDuplicatesSpark, Sambamba and FastDup runtime for four input sizes. (f) Picard MarkDuplicates, GATK MarkDuplicatesSpark, Sambamba and FastDup peak memory usage for four input sizes. (g) Picard MarkDuplicates, GATK MarkDuplicatesSpark, Sambamba and FastDup thread scalability.

FastDup requires coordinate-sorted SAM/BAM files, where reads are ordered by their mapped genomic positions. However, due to read clipping during alignment, the actual positions used for duplicate detection may differ from the recorded mapping positions. Thus, sorting of all reads is required before duplicate identification. As shown in Fig. 1a, the marking duplicates workflow of conventional tools comprises four sequential steps: (i) Data Reading and Decompression: Reading and decompressing each data block into memory (only a subset of data can be loaded into memory each time due to the large size of the input file). (ii) Generating read keys and finding read pairs: Creating unique keys for each read and identifying read pairs within each block until all the data blocks in the input file are processed. (iii) Global sorting: Sorting all the paired and unpaired reads. (iv) Checking duplicate: Detecting duplicate reads within the entire data. Conventional workflows require completing the first two steps across all data before initiating subsequent steps. Given typical input sizes, intermediate data from step 2 must be stored externally. This combination of temporary files and global sorting creates significant performance bottlenecks.

First, FastDup circumvents global sorting through a novel speculation-and-test workflow as shown in Fig. 1b. This approach is based on the following two observations: (i) Clipping is not common in the mapped reads, which means most reads retain original mapping positions for duplicate detection. (ii) Read pairs typically reside near each other in coordinate-sorted files. We introduce Test-overlap step to implement the speculation-and-test workflow, which operates as follows: (i) Speculation Phase: Initially assuming data blocks are independent and processing each data block through the full pipeline. (ii) Test Phase: Resolving inter-block dependencies and correcting misclassified duplicates during Test Overlap. This approach eliminates global sorting while maintaining accuracy.

Second, we make performance profiling on the five subtasks and find that the first three subtasks account for over 90% of total execution time as shown in Fig. 1c. This finding prompted our adoption of data parallelism for acceleration. According to user-specified thread parameters, we further partition data blocks into sub-blocks distributed across worker threads. Each thread processes its assigned sub-block through decompression, read keys generation, and sorting stages. This data-parallel approach reduces execution time linearly with respect to the thread parameter for the first three tasks.

Finally, to further improve performance, FastDup uses a parallel pipeline architecture that leverages task-parallel across different data blocks. As shown in Fig 1d: compared to traditional serial pipelines, this parallelized pipeline achieves over 50% speedup.

Additionally, FastDup supports duplicate classification, distinguishing between PCR duplicates and optical duplicates. It also generates a metrics file containing detailed statistics on duplicate occurrences, providing valuable insights for downstream analysis.

## 3 Results

To evaluate the correctness and performance improvements of FastDup, we compare it against three widely used tools: Picard MarkDuplicates (Broad Institute 2025), GATK MarkDuplicatesSpark (Van der Auwera and O’Connor 2020), and Sambamba (Tarasov *et al.* 2015). For each tool, we recorded runtime and peak memory usage across multiple datasets. All input BAM files are publicly available through the NCBI (NCBI Homepage: https://www.ncbi.nlm.nih.gov.) (2025) Sequence Read Archive (SRA).

Table 1 summarizes the characteristics of the four datasets used in the evaluation, which include both whole-exome sequencing (WES) and whole-genome sequencing (WGS) data. The number of reads per dataset ranges from 100 million to 1.58 billion. These BAM files were generated using the BWA-MEM aligner. All tools, including FastDup, produced identical duplicate marking results, with the total number of marked duplicates shown in the final column of Table 1.

**Table 1.** Four experimental human datasets (NCBI 2025).

Source	Type	Length (bp)	No. of reads	No. of duplicates
SRR25735654	WES	145	118 205 082	13 019 231
SRR8381428	WES	148	283 703 444	59 461 068
SRR25735656	WGS	151	1 069 743 600	180 186 251
ERR194147	WGS	101	1 582 771 014	23 855 846

Figure 1e presents the wall time required by each tool to complete duplicate marking across the four datasets. The results demonstrate that: (i) FastDup achieves an average speedup of 20.13×, 8.03×, and 4.56× over Picard MarkDuplicates, GATK MarkDuplicatesSpark, and Sambamba, respectively. (ii) FastDup maintains stable performance across datasets of varying sizes, showing scalability independent of input volume.

Figure 1f shows the peak memory usage of all tools. The observations are as follows: (i) GATK MarkDuplicatesSpark exhibits the highest memory usage, requiring large memory allocations regardless of dataset size. (ii) Picard MarkDuplicates and Sambamba use less memory, but their memory consumption scales with the input size. (iii) FastDup consistently consumes the least memory among all tools, even when processing the largest datasets.

Figure 1g shows the thread scalability of all tools. We can find that: (i) FastDup consistently outperforms baselines across all thread numbers. (ii) FastDup demonstrates near-linear scalability up to 16 threads. (iii) When the number of threads exceeds 16, the performance gains of FastDup become limited due to constraints such as disk I/O throughput.

All experiments were conducted on a 32-core AMD server equipped with an AMD 3970X processor and 128 GB of RAM. Picard MarkDuplicates and GATK MarkDuplicatesSpark were executed using OpenJDK 19.0.2, with a maximum heap size set via -Xmx100G. All input and output files were in BAM format. For tools supporting multithreading, the number of threads was set to 32.

## 4 Discussion

Our evaluation shows that FastDup produces identical results to Picard MarkDuplicates while achieving up to a 20-fold increase in performance. This improvement stems from FastDup’s ability to exploit the structure of coordinate-sorted BAM files—specifically, the observation that most paired reads are located near each other and are approximately sorted by position. As a result, FastDup eliminates the need for expensive global sorting operations. This insight enables finer-grained parallelism: FastDup divides the input into small, independent data blocks, processes each in parallel using a speculation-and-test strategy, and subsequently resolves cross-block dependencies. The use of a parallel pipeline architecture further enhances throughput and overall efficiency.

Picard MarkDuplicates (Broad Institute 2025), GATK MarkDuplicatesSpark (Van der Auwera and O’Connor 2020), and Sambamba (Tarasov *et al.* 2015) are widely used tools for duplicate marking. While they offer accurate results, all three rely heavily on global coordinate sorting, which leads to significant memory usage. When memory is insufficient, intermediate data must be written to disk, further impacting performance. Additionally, their parallel scalability is limited, making them less suitable for high-throughput or large-scale datasets. These challenges are common limitations across traditional duplicate marking tools.

In contrast to traditional tools, streaming-based approaches such as Samblaster (Faust and Hall 2014) and streammd (Leonard 2023) offer real-time duplicate marking by integrating into the mapping pipeline. They utilize lightweight data structures like hash tables or Bloom filters to detect duplicates on the fly. However, these methods do not offer the same level of precision: they may miss certain duplicates or retain suboptimal reads, as they lack access to global sorting information. While faster than traditional tools, their reduced accuracy limits their suitability for high-precision workflows.

FastDup combines the accuracy of traditional tools with the performance benefits of streaming approaches. Its speculation-and-test mechanism allows for fine-grained parallelism while maintaining exact duplicate marking results identical to those of Picard MarkDuplicates (Broad Institute 2025). However, it currently only supports coordinate-sorted SAM/BAM input files. FastDup supports seamless integration into the GATK (Van der Auwera and O’Connor 2020) genomic analysis pipeline.

## Data Availability

WES and WGS data used in the performance evaluation are publicly available through the NCBI Sequence Read Archive (SRA). The SRA accession IDs are listed in Table 1.

## References

[btaf633-B1] Bonfield JK , Marshall J, Danecek P et al HTSlib: C library for reading/writing high-throughput sequencing data. Gigascience 2021;10:11–6. 10.1093/gigascience/giab007 PMC7931820 33594436

[btaf633-B2] Faust GG , Hall IM. SAMBLASTER: fast duplicate marking and structural variant read extraction. Bioinformatics 2014;30:2503–5. 24812344 10.1093/bioinformatics/btu314 PMC4147885

[btaf633-B3] GitHub Repository. Broad Institute. Picard Toolkit. 2025. https://broadinstitute.github.io/picard/ (1 March 2025, date last accessed).

[btaf633-B4] Leonard C. streammd: fast low-memory duplicate marking using a bloom filter. Bioinformatics 2023;39:181–4. 10.1093/bioinformatics/btad181 PMC10112951 37027230

[btaf633-B5] NCBI. NCBI Homepage. 2025. https://www.ncbi.nlm.nih.gov (1 March 2025, date last accessed).

[btaf633-B6] Tarasov A , Vilella AJ, Cuppen E et al Sambamba: fast processing of NGS alignment formats. Bioinformatics 2015;31:2032–4. 25697820 10.1093/bioinformatics/btv098 PMC4765878

[btaf633-B7] Van der Auwera GA , O’Connor BD. *Genomics in the Cloud: Using Docker, GATK, and WDL in Terra*, 1st edn. United States of America: O'Reilly Media, Inc., 2020.

